# Writing by proxy: Authentic authorship in the era of AI‐powered communication of scientific knowledge

**DOI:** 10.1002/ccs3.70120

**Published:** 2026-10-15

**Authors:** Brahim Chaqour

**Affiliations:** ^1^ Department of Molecular Ophthalmology Perelman School of Medicine University of Pennsylvania Philadelphia Pennsylvania USA

**Keywords:** analytical tools, artificial intelligence, copyediting, disclosure, grant proposal, intellectual ownership, publication

## Abstract

Artificial intelligence (AI) is reshaping biomedical research as both a key analytical tool and an efficient copyeditor. Large language models and related algorithms can help investigators sift through the ever‐expanding bodies of scientific literature, connect fragmented biological and clinical datasets, and uncover/recognize meaningful patterns buried within complex multidimensional data. Beyond the bench, these tools can improve and refine manuscript architecture and streamline the flow of information by honing the articulation of simple or complex scientific ideas and sharpening the clarity and cadence of scientific information, which ultimately strengthens the writing in publications and grant proposals alike. For researchers working across languages or at early career stages, these tools allow them to express ideas with greater clarity and fluency, ultimately ensuring that intellectual contributions are assessed on the basis of their conceptual merit and experimental validity. However, the growing persuasiveness of AI‐generated writing raises an important concern as to whether persuasive language can create an impression of rigor that is not necessarily matched by rigorous science. AI tools can improve the clarity and presentation of scientific writing, but they cannot establish biological relevance, experimental validation and/or the nuanced judgment required to identify confounding variables, and draw meaningful conclusions. Similarly, an eloquently written grant proposal cannot substitute for an innovative idea, testable hypothesis, feasible experimental approach, and meaningful anticipated contribution. It is therefore essential to view AI tools as instruments of improvement not replacement. They can enhance clarity, efficiency, and readability but they should not replace investigators' intellectual ownership, accountability, and critical interpretation. Disclosure of AI use, together with continued investigators' responsibility for originality, accuracy, citations, data integrity, and interpretation, is essential to maintain trust in scientific discoveries. Similarly, editors, peer reviewers, authors, and funding panels bear a renewed obligation to distinguish writing quality from scientific quality and continue to evaluate approach, innovation, experimental rigor, reproducibility, investigator skills, and scientific and/or clinical significance. Used judiciously and with discernment, AI can free researchers to invest more deeply in the irreplaceable human dimensions of scientific discovery.

## Graphical abstract

Evolution of AI as an analytical tool and an efficient copyeditor. AI use in 2015, you wrote and discussed what you investigated and found. By 2025, AI helps with the drafting, the editing, and much of the analysis. Efficiency is undeniable. By 2029, fluency will likely be free but the hardest questions still belong to the human investigators such as what is worth asking and which finding survives checking.

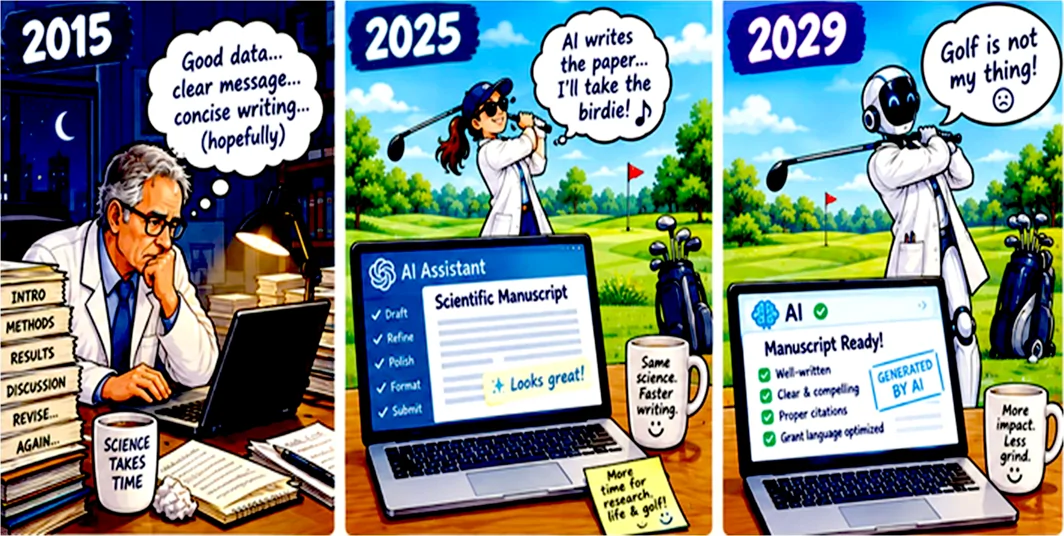

Artificial intelligence (AI) systems also known as large language models are trained on massive text datasets to understand, generate, and manipulate human language with remarkable fluency. AI tools show promise for extracting patterns from large datasets, combining and curating heterogeneous datasets, literature mining, organizing clinical documentation, extracting structured data from electronic health records, and supporting diagnostic reasoning among many other things.^1^, ^2^ When AI tools are used to improve grammatical accuracy, sentence structure, and overall clarity, and readability of scientific communications, publications, and grant proposals, the time‐to‐first draft can drop substantially, enabling researchers from nonnative English‐speaking countries and/or early stage investigators to improve their writing style and publish confidently and potentially more frequently.^3^, ^4^ AI tools can further assist with argument polishing, logical flow, and stylistic consistency and ensure that manuscript sections, for instance, align with a journal's guidelines. As of 2026, virtually every major scientific publisher, including JCCS publisher Wiley, agrees on the following three points for handling AI use in manuscripts, although the details vary by publisher:^5^ (1) no AI authorship, (2) mandatory disclosure of any and/or substantive use of AI, and (3) full authors' accountability for manuscript content. Similarly, when writing grant proposal, AI tools can help organize Specific Aims, articulate better innovation statements, and polish significance and research plan sections, although the core ideas must remain the PI's own as per the guidelines of funding agencies such as NIH (NIH Notice NOT‐OD‐25‐132).

Diverse AI algorithms are available to researchers and even more refined and improved ones are likely in the work. As a result, AI has sprinted from a science‐fiction sidebar just a few years ago to a daily lab‐bench/office‐desk partner, quietly but surely reshaping how studies and experiments are designed, executed, and communicated/written. However, the ethical use of AI in scientific writing is less about the tool and more about the task.^6^ Grammar and spelling checks, readability tweaks, translation of text, and formatting references are generally deemed to be an acceptable use of it. De novo drafting of manuscripts and grant applications, primary data interpretation, literature reviews, and plagiarism checks with no robust human verification are ethically problematic. As AI tools become more fluent than humans in the language of grants and publications, a new kind of AI use‐related anxiety is brewing, that is, is AI empowering researchers to write manuscripts and well‐crafted documents or enabling the generation of “smart‐sounding” writings whose true intellectual parents are AI tools?

On one side stood the more traditional researchers who have long wrestled with their own sentences, arguments, and creative expressions while drafting, editing, and revising manuscripts and grant proposals. Some regarded AI primarily as an advanced spellchecker or stylistic aid. Their writing process may be slower but it is closely tied to the development and articulation of ideas that are, for better or for worse, their own. On the other side is the community of scientists of all ages who came to realize that AI tools can substantially enhance many aspects of scientific document development and presentation. Beyond their copyediting prowess, AI systems can integrate and interrogate large, heterogeneous datasets such as genomic, transcriptomic, proteomic, metabolomic and acetylomic profiles, single‐cell RNA‐sequencing data, published literature, drug databases, and clinical and preclinical phenotypical data and other processes. Such integration can reveal unexpected relationships, generate new hypotheses, and help prioritize potentially important biological or therapeutic questions. Nevertheless, these outputs are not new discoveries but simply hypotheses or associations until their biological relevance has been rigorously examined through appropriate experimental validation or demonstrated in well‐designed preclinical and clinical studies.

The same tension extends to grant proposal development. AI can support not only language editing but also literature searching and organization, preliminary synthesis of information, and refinement of individual proposal components. It is therefore understandable that investigators may wonder why they should labor to produce a cumbersome Specific Aims page on their own when a well‐trained AI model can rapidly generate prose that is clearer, more concise, and seemingly more responsive to reviewers' expectations. The greater temptation is to outsource not only writing but also the intellectual work of brainstorming, data interpretation, and experimental planning and then present the resulting output as one's own “oeuvre d'art” or “chef‐d'oeuvre.” This is where the distinction between assistance and authorship becomes essential. AI may substantially improve the organization and presentation of scientific knowledge, but it cannot guarantee that a proposed hypothesis is biologically sound, that an experimental design is feasible and appropriately controlled, or that an anticipated result will be reproducible. AI‐generated content may also contain inaccurate interpretations, overlooked limitations and confounding variables, unsupported claims, fictive references, and recommendations that are inappropriate for the biological or clinical context. Accordingly, investigators must retain full intellectual ownership and critically verify every AI‐assisted element in a publication or a grant proposal. Only if used responsibly, this capability can help investigators communicate ambitious ideas more effectively and devote more time to refining the underlying scientific rationale, experimental design, and anticipated results.

That said, AI is not the enemy of authentic authorship but unchecked reliance on it can be. Major scientific publishers agree that journals should require authors to disclose whether AI tools were used to produce their submitted work and, if any, how they were used. At all times, authors must carefully check the content of their manuscript and remain responsible for it. Journals are increasingly using AI to screen submitted manuscripts for formatting compliance and AI content, assist with identifying potential reviewers, and shape publishing workflows. Editors and reviewers, who are also authors of their own works, have a responsibility to determine whether a manuscript meets both the standards of language and those of authentic human reasoning. Who knows? As AI tools continue to advance rapidly, future systems may also assist in evaluating these aspects albeit imperfectly. Similarly, grant proposal review faces these very challenges. As a result, the true scientific merit of a grant application may be decided only retrospectively, after it has been funded and the proposed work has been completed and its output can be assessed. In his study of LLMs and the direction and impact of US federal research funding, Qian et al. found that higher AI involvement predicts greater publication output though concentrated in nonhit papers rather than the most highly cited studies.^7^ This reality further underscores the importance of manuscript peer review, where the significance and reliability of a research study is carefully assessed through human reasoning.

All in all, researchers are likely to benefit most from using AI as a practical tool to analyze big data, clarify ideas, improve expression, and streamline routine aspects of writing while preserving and cultivating the habits that underpin good science including reading, reasoning thoughtfully, writing independently, and revising carefully. The goal is not to emulate or outperform current or future AI tools but to use them in ways that strengthen scientific communication and support more focused, creative, and rigorous research.

## AUTHOR CONTRIBUTIONS

*Drafted and revised this editorial*: Brahim Chaqour.

## CONFLICT OF INTEREST STATEMENT

Dr. B. Chaqour is the Editor‐in‐Chief of the Journal of Cell Communication and Signaling.

## ETHICS STATEMENT

Not applicable.

## CONSENT FOR PUBLICATION

Not applicable.

## Data Availability

Data sharing not applicable to this article as no datasets were generated or analyzed during the current study.
